# Alcohol Facilitates CD1d Loading, Subsequent Activation of NKT Cells, and Reduces the Incidence of Diabetes in NOD Mice

**DOI:** 10.1371/journal.pone.0017931

**Published:** 2011-04-01

**Authors:** Karsten Buschard, Axel Kornerup Hansen, Karen Jensen, Dicky J. Lindenbergh-Kortleve, Lilian F. de Ruiter, Thomas C. Krohn, Majbritt R. Hufeldt, Finn K. Vogensen, Bent Aasted, Thomas Osterbye, Bart O. Roep, Colin de Haar, Edward E. Nieuwenhuis

**Affiliations:** 1 Bartholin Instituttet, Rigshospitalet, Copenhagen, Denmark; 2 Department of Veterinary Disease Biology, Faculty of Life Sciences, University of Copenhagen, Frederiksberg, Denmark; 3 Laboratory of Pediatrics and Department of Pediatric Gastroenterology, Erasmus MC, Rotterdam, The Netherlands; 4 Department of Food Sciences, Faculty of Life Sciences, University of Copenhagen, Frederiksberg, Denmark; 5 Department of Immunohaematology and Blood Transfusion, Leiden University Medical Center, Leiden, The Netherlands; 6 Department of Gastroenterology and Hepatology, Erasmus MC, Rotterdam, The Netherlands; 7 Department of Paediatric Gastroenterology, Wilhelmina Children's Hospital, University Medical Centre Utrecht, Utrecht, The Netherlands; University of Bremen, Germany

## Abstract

**Background:**

Ethanol (‘alcohol’) is a partly hydrophobic detergent that may affect the accessibility of glycolipids thereby influencing immunological effects of these molecules.

**Methods:**

The study included cellular *in vitro* tests using α-galactosylceramide (αGalCer), and *in vivo* NOD mice experiments detecting diabetes incidence and performing behavioural and bacterial analyses.

**Results:**

Alcohol in concentrations from 0.6% to 2.5% increased IL-2 production from NKT cells stimulated with αGalCer by 60% (p<0.05). CD1d expressed on HeLa cells contained significantly increasing amounts of αGalCer with increasing concentrations of alcohol, suggesting that alcohol facilitated the passive loading of αGalCer to CD1d. NOD mice were found to tolerate 5% ethanol in their drinking water without signs of impairment in liver function. Giving this treatment, the diabetes incidence declined significantly. Higher numbers of CD3+CD49b+ NKT cells were found in spleen and liver of the alcohol treated compared to the control mice (p<0.05), whereas the amount of CD4+Foxp3+ regulator T cells did not differ. Increased concentrations of IFN-γ were detected in 24-hour blood samples of alcohol treated mice. Behavioural studies showed no change in attitude of the ethanol-consuming mice, and bacterial composition of caecum samples was not affected by alcohol, disqualifying these as protective mechanisms.

**Conclusion:**

Alcohol facilitates the uptake of glycolipids and the stimulation of NKT cells, which are known to counteract Type 1 diabetes development. We propose that this is the acting mechanism by which treatment with alcohol reduces the incidence of diabetes in NOD mice. This is corroborated by epidemiology showing beneficial effect of alcohol to reduce the severity of atherosclerosis and related diseases.

## Introduction

Moderate intake of ethanol (‘alcohol’) has epidemiologically been shown to have a beneficial effect on longevity [1], [2]. Alcohol consumption in lower doses reduces the risk of cardio-vascular diseases, probably in part through effects on lipids and haemostatic factors [3]. Even the risk for developing Type 2 diabetes is reduced although no clear-cut mechanism has been established [4]. High alcohol consumption results in development of insulin resistance [5], and a U-shaped association indicates that moderate alcohol consumption is associated with the highest insulin sensitivity [6]. In rats, acute administration of ethanol causes insulin resistance in a dose-dependent manner [7]. Moderate alcohol consumption has been associated with lower levels of inflammatory markers [8]–[10], but no studies have described effects on the classical innate immune system.

CD1d molecules load various glycosphingolipids (in laboratory studies often α-galactosylceramide (αGalCer)) which presentation stimulates NKT cells [11], [12]. By means of IL-2 production these CD1d-restricted NKT cells are then able to stimulate regulator T cells [13]. Glycosphingolipids are composed of ceramide with fat chains of various length and saturation, and of different sugars, often galactose. Furthermore, sulphate, sialic acid or other structures can be attached. Since alcohol itself is a detergent dissolving fat components in a hydrophilic milieu, it might theoretically facilitate the loading of the two fat chains of the glycosphingolipids to the CD1d molecules into the pockets designed for this [12]. If confirmed, a beneficial effect on diabetes development in NOD mice might be conceivable, since treatment with the glycosphingolipids of both αGalCer [14], [15] and sulfated βGalCer (sulfatide) [16], have been shown to reduce diabetes incidence.

Therefore, the question raises whether alcohol in any way could influence the uptake of αGalCer, and whether alcohol might affect the pathogenesis of Type 1 diabetes (T1D). Furthermore, is there a connection between alcohol and the regulatory T cells and NKT cells, and does alcohol influence the gut microbiota, which is known to affect T1D development [17]?

## Materials and Methods

### *In vitro* studies

#### αGalCer experiments and IL-2 production

Epithelial cell-line T84d [18] (1*10^5^ cells/ml) was loaded with 10 ng/ml αGalCer in the presence of 0.0, 0.3, 0.6, 1.3, 2.5, 5.0% ethanol. After 18 h of incubation the epithelial cells where washed 3 times with medium and NKT cells (DN32.D3) were added in a concentration of 5*10^5^ cells/ml. After 24 h of culture the supernatant was collected, and IL-2 production was determined by ELISA.

#### Uptake of ^3^H-αGalCer

HeLa cells and CD1d transfected HeLa-D cells, respectively, were maintained at 37°C, 5% CO_2_ in DMEM (Lonza, Basel, Switzerland) supplemented with 10% FCS (Lonza), non-essential amino acids (NEAA) (Sigma-Aldrich, St. Louis, MO) and pen-strep (Lonza). For labelling experiments, 100,000 cells/well were plated in 96 well tissue culture plates (Nunc, Roskilde, Denmark) in culture media and allowed to attach. Then alcohol (0–5%) and ^3^H-αGalCer (30 nmol/ml) was added and allowed to incubate 18 h at 4°C. Cells were washed 3 times in cold PBS, mixed, harvested and lysed in Milli-Q water using a Cell Harvester; cell lysate was mixed with scintillation liquid (Optiphase Hisafe2, PerkinElmer, Shelton, CT) and ^3^H activity was measured in a scintillation counter (Tri-Carb 1600 TR, Packard, Meriden, CT).

### Animals

#### Evaluation of ethanol doses for animals

In a pre-test study 15 female NOD/Bom mice (Taconic Europe, Ry, Denmark), eight weeks old, were given 2.5, 5 or 10% v/v ethanol in the drinking water. After two weeks the mice were sacrificed and blood from heart puncture was collected and analysed for the concentration of bilirubin and various liver enzymes and for ethanol.

#### Diabetes incidence study

Forty female NOD/Bom mice were from the age of 3 weeks housed in groups of four to six in type 3 macrolon cages (Tecniplast, Buguggiate, Italy) with aspen bedding and environmental enrichment (Tapvei, Korteinen, Finland). The temperature was 21°C, the light was on from 6:00 am–6:00 pm and the air was changed 10–12 times/hour. Alcohol was given to 21 mice from the age of six weeks in the drinking water in 5% v/v on the basis of the pre-test study (see above), whereas 19 mice were controls similarly treated but without alcohol. All mice were weighed three times weekly. Mice losing weight from day to day were urine sampled (Multisticks SG, Bayer AG, Leverkusen, Germany), and if urine contained glucose, blood was sampled from the tail vein and tested for glucose (Ascension Elite and Glycostics, Bayer AG). Any mouse with a blood glucose concentration equal to or above 12 mmol/l was considered diabetic and was then sacrificed. For this the mice were anaesthetized by fentanyl-fluanisone-midazolam, bled, and euthanized by cervical dislocation (Flecknell PA. Laboratory Animal Anaesthesia (2nd edn). Academic Press: London, 1996). All housing and maintenance principles were in accordance with the Council of Europe Convention ETS 123 (European Convention for the Protection of Vertebrate Animals used for Experimental and other Scientific Purposes (ETS 123). Council of Europe: Strasbourg, 1986) and were equivalent to the PHS Policy on Humane Care and Use of Laboratory Animals (Public Health Service. PHS Policy on Humane Care and Use of Laboratory Animals. Office of Laboratory Animal Welfare: Bethesda, MD, 2004).

#### Immunological studies

Thirty-two NOD/Bom mice were classified in four groups. Group one and two had 5% ethanol v/v added into their drinking water for two weeks, and 24 hours before euthanation group one had αGalCer and group two vehicle injected intraperitoneal. Group three and four had drinking water without ethanol the two weeks, and 24 hours before euthanation group three had αGalCer and group four vehicle injected intraperitoneal. After the injections blood samples were drawn two and six hours later from the lateral tail vein, and 24 hours later from the periorbital vein plexus. The blood samples were centrifuged and plasma isolated. The mice were euthanized by cervical dislocation, and the spleen, liver, mesenterial lymph nodes and Peyer's patches were isolated. After single cell preparation all four organs had CD4 and Foxp3 antibodies added, spleen and liver CD3 and CD49b antibodies added, and spleen CD1d antibody added which subsequently were analysed by means of FACS. Plasma from the two-hour blood samples were analysed for TNF-α, IFN-γ, and IL-2 by means of Cytometric Bead Array (CBA), while the plasma from the 24-hour blood samples were analysed for IFN-γ by means of ELISA and spectrophotometry.

#### Behavioural tests

All the forty mice from the incidence study were tested twice in an open field test with an arena diameter of 90 cm and an inner circle of 40 cm for 10 min and in an elevated plus maze (50×50 cm, height 50 cm) for 10 min; once at the age of 11 weeks before onset of diabetes, and again the day after they had been tested diabetic. All the behavioural studies were performed between 8:00 and 10:00 am. Test data were analysed by the use of Ethovision (Noldus Information Technology, The Netherlands) and the distance moved (in cm) and the duration (in sec) in each zone for each mouse were calculated.

### Histochemistry

Four µm paraffin sections were routinely stained with hematoxylin (Vector Laboratories, Burlingame, CA) and eosin (Sigma-Aldrich, Zwijndrecht, The Netherlands) to study morphological alterations. For immunohistochemistry sections were deparaffinised and endogenous peroxidases were quenched with 3% H_2_O_2_ in methanol for 20 min. Antigen retrieval was obtained after microwave treatment in citrate buffer (10 mM pH 6.0). Sections were blocked for 1 hour in either 1% blocking reagent (Roche, Almere, The Netherlands) in PBS or 10 mM Tris, 5 mM EDTA, 0.15 M NaCl, 0.25% gelatine, 0.05% Tween-20, 10% normal mouse serum, 10% normal rabbit serum, pH 8. Antibody incubation was performed overnight at 4°C with rabbit anti-CD3 (Dako, Heverlee, Belgium) diluted 1∶800 and anti-Foxp3 (clone FJK-16s, eBioscience, San Diego, CA) using a dilution of 1∶100. Immunoreactions were detected using respectively biotinylated secondary goat-anti-rabbit or rabbit-anti-goat serum with the Vectastain ABC Elite Kit (Vector Laboratories) and 3,3′-diaminobenzidine tetrahydrochloride (Sigma-Aldrich). Nuclei were counterstained with Hematoxylin (Vector Laboratories). H&E-stained pancreatic sections were used to score the insulitis as follows: G0, no inflammation; G1, peri-insulitis but no intra-insulitis; G2, 0–50% intra-insulitis; G3, more than 50% intra-insulitis [14].

### Caecum samples for denaturating gradient gel electrophoresis (DGGE)

Caecum samples were collected from all forty mice in the incidence study. DNA was immediately extracted from samples using the QIAamp DNA Stool Mini Kit (Qiagen, Hilden, Germany) according to the manufacturer's instructions. The DNA was amplified by means of Polymerase Chain Reaction (PCR), using primers specific to the V3 region of 16S rDNA. Amplicons were thereafter analyzed by means of DGGE using an acrylamide gel containing a 30%–65% chemical gradient (urea and formamide) that enabled separation of the DNA amplicons based on sequence differences in the V3 region. PCR and DGGE conditions are lined out in detail elsewhere [19]. Data analysis comparing band patterns of DGGE profiles was done in Bionumerics Version 4.5 (Applied Maths, Sint-Martens-Latem, Belgium). Dendrogram and three-dimensional principle component analysis (3D-PCA) derived from the similarity of the DGGE patterns, based on Dice's coefficient of similarity with the unweighted pair group method with arithmetic averages clustering algorithm, were constructed.

### Statistics

Biochemical values were shown as mean ± SEM and compared and analysed using Students t-tests. Accumulative diabetes incidence was examined by Kaplan-Meier estimation of which statistical significance was evaluated by the log-rank test. The behavioural results were analysed by use of 2-sample t-test (Minitab ver. 14, Minitab Inc.). Data from the immunological studies were analysed by use of one-way ANOVA and two-sample t-test. When no normality was found, Kruskal Wallis test was used (Minitab ver. 15). The level of significance was set at p<0.05.

## Results

### *In vitro* study of alcohol and CD1d loading

**Figure 1:**
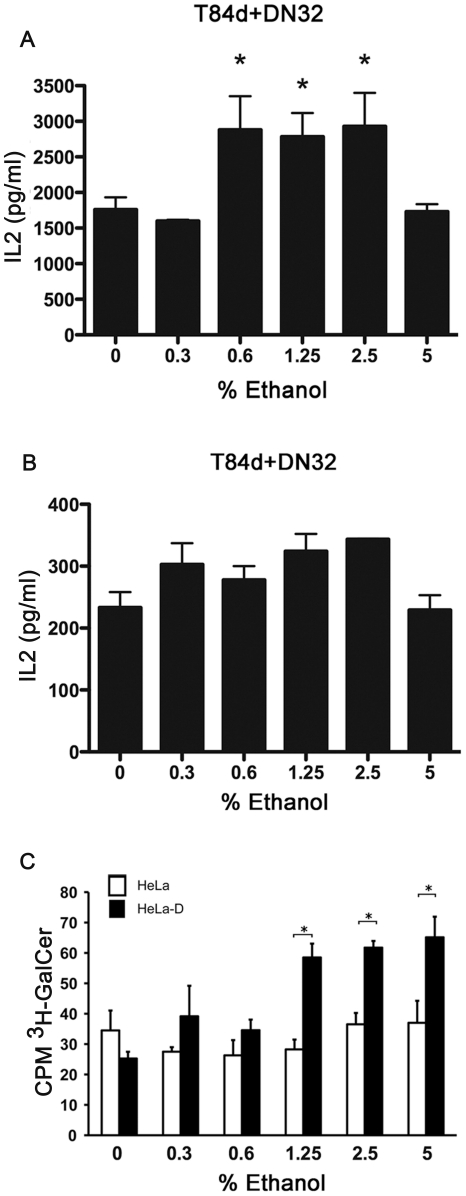
Low levels of ethanol stimulate activation of NKT cells via enhanced CD1d antigen loading. A) T84d cells were loaded with αGalCer in combination with different concentrations of ethanol. The next day these cells were incubated with DN32 NKT cells and the production of IL-2 was measured after 24 hours. B) T84d cells were loaded with αGalCer. The next day these cells were incubated with DN32 NKT cells in combination with different concentrations of ethanol. The production of IL-2 was measured after 24 hours. C) Uptake of ^3^H-αGalCer in HeLa cells (open bars) and HeLa-D cells (solid bars) exposed to ethanol in the range 0–5% at 4°C shows a positive correlation to alcohol concentration. Error bars indicate SEM, *p<0.05.

First, T84d epithelial cells incubated with αGalCer in various concentrations of alcohol. Eighteen hours later the cells were washed and added to DN32.D3 NKT cells. After 24 h, IL-2 production was measured as endpoint. Between 0.6% and 2.5% ethanol the IL-2 concentration was significantly increased (p<0.05) compared to control incubation without alcohol (Fig. 1A). Ethanol at 0.3% showed no effect (Fig. 1A). Next, we performed similar experiments with the exception that alcohol was added at the time of NKT cell addition, rather then during αGalCer loading. Although 0.3% ethanol induced some minor increase in IL-2 production (ns), the overall levels were less than those seen in the initial experiments (Fig. 1B). Thus, alcohol incubation during activation of CD1d restricted antigen presentation enhanced NKT activation most likely due to facilitation of CD1d loading. This was seen in all five experiments performed. The optimal ethanol concentration varied from 0.5 to 5.0%.

Next, we turned to look at the absolute uptake of ^3^H-labelled glycolipid (αGalCer) in HeLa cells and HeLa cells expressing CD1d (HeLa-D), respectively, during exposure to alcohol (Fig. 1C). To investigate the passive loading of glycolipid to CD1d, we labelled viable but metabolic inactive cells at 4°C. Metabolic inactive HeLa cells were not labelled, while increasing concentrations of alcohol had no effect. HeLa cells expressing CD1d (HeLa-D) was found to contain increasing amounts of ^3^H-αGalCer with increasing concentrations of alcohol (p<0.01) suggesting that alcohol facilitated the passive loading of αGalCer to CD1d.

### Dose-response study on alcohol treatment of mice

The effect of consumption of different doses of ethanol to NOD mice was first evaluated. The clinical biochemistry data from the groups of 2.5 and 5.0% doses of ethanol were almost similar. In contrast, for the group of mice receiving 10% alcohol the level of basic phosphatase was decreased (74±9 vs. 113±5 U/l, p = 0.01) and lactate dehydrogenase concentration increased (280±29 vs. 182±15 U/l, p = 0.03) compared to mice having lower doses. The concentration of bilirubin and alanine aminotransferase (ALAT) tended both to be higher for the high dose ethanol group whereas there was no difference for the concentration of albumin and serum protein as well as the concentration of blood glucose. Interestingly, compared to the estimated daily intake of alcohol, which was 600 mg/mouse for the 10% group, the blood concentration of ethanol was surprisingly low and equal for the two lowest alcohol groups (0.84±0.04 mmol/l), only showing a tendency for increases in the high ethanol consumption group (1.43±0.59 mmol/l, ns). In order to avoid biochemical abnormalities related to the liver, the highest dose of alcohol consumption not showing this was chosen for the incidence studies, *i.e.*, 5% v/v ethanol intake.

### Diabetes incidence is reduced in NOD mice treated with ethanol

**Figure 2:**
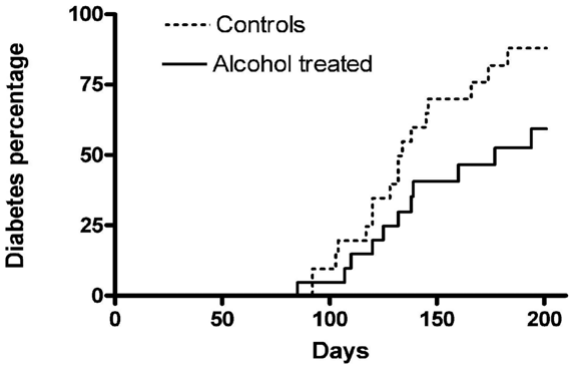
Ethanol reduces diabetes incidence in NOD mice. Kaplan-Meier curves showing the cumulative incidence of diabetes in alcohol treated (firm line) and in control NOD/Bom mice (dotted line). Log-rank test evaluated p<0.05.

Starting from 6 weeks of age until age 28 weeks the incidence of diabetes was recorded in NOD mice treated with 5% v/v ethanol compared to a control group receiving only pure drinking water. Diabetes began to develop quite similarly in the two groups, but reached a significantly lower level of 64% in the ethanol group compared to 89% in the control group (p<0.05; Fig. 2). The blood glucose level at diagnosis of diabetes was 17.4±0.7 mmol/l for the ethanol group compared to 19.4±0.8 mmol/l for the control group, which was significantly different (p<0.05).

To assess whether this reduced diabetes incidence was due to reduced T cell infiltration into the islets, we scored the degree of insulitis. Although no significant differences could be detected between the ethanol and control groups, our data show reduced insulitis score in ethanol treated mice, in which we found more islets without insulitis and fewer islets with peri-insulitis only or with less than 50% intra-insulitis compared to the control mice.

### Flow cytometry and cytokines

Significantly higher numbers of NKT cells (CD3+CD49b+ cells) were found in the spleens (p<0.05) and the livers (p<0.05) of alcohol treated mice compared to the control mice (Table 1). No significant differences were detected in the numbers of regulatory T cells (CD4+Foxp3+ cells) in the spleen, liver, mesenterial lymph nodes or Peyer's patches in the alcohol treated mice compared to control mice. No significant differences were detected in the concentration of the cytokines in the two-hour blood samples in relation to alcohol treatment, whereas a significantly higher concentration (p<0.05) of IFN-γ were detected in the 24-hour blood samples of alcohol treated mice (Table 1). No significant differences were detected in the numbers of CD3+CD49b+cells in the spleens and livers or in the concentration of INF-γ in the two-hour blood samples in αGalCer treated mice compared to control (vehicle) mice. In contrast, a higher amount of TNF-α (12.2±1.6 vs. 9.4±0.9 pg/ml, p<0.05) and IL-2 (1.6±0.1 vs. 1.4±0.1 pg/ml, p<0.05) were found in the two-hour blood samples in αGalCer treated mice compared to control (vehicle) mice.

**Table 1:** NOD mice with 5% ethanol v/v in the drinking water had a significantly higher number of NKT cells (CD3+CD49b+ cells) in both liver and spleen and a significantly higher concentration of IFN-γ in their 24 hour blood samples compared to NOD mice without ethanol in their drinking water.

	1	2	3	4
Group	Alcohol+αGalCer	Alcohol+vehicel	No alcohol+αGalCer	No alcohol+vehicel
Alcohol vs no alcohol:				
**Percent CD3+CD49b+cells**				
Spleen				
n	8	7	7	6
Mean ± SEM	3.06±0.34	2.83±0.31	2.57±0.21	2.20±0.90
p	Group 1+2 vs 3+4: p<0.05			
Liver				
n	7	7	8	5
Mean ± SEM	4.07±0.42	5.09±0.29	3.94±0.37	3.94±0.38
p	Group 2 vs 4: p<0.05			
**Pg/ml INF-γ**				
24 hours blood				
n	6	-	7	-
Mean ± SEM	6506±727		3973±838	
p	Group 1 vs 3: p<0.05			

### Behavioural studies

Alcohol had no impact on the behaviour of the mice in the elevated plus maze. When mice became diabetic they moved less and spent less time on the open arms (p<0.001). When testing the animals for an effect of alcohol in the open field test, the control group moved longer around in the outer circle after onset of diabetes (p<0.05), but except for this no differences could be found between the alcohol group and the control group. After onset of diabetes the mice changed behaviour and became more anxious and moved less around in the centre of the circle (p<0.05). For the distance moved in the outer circle, no differences could be detected.

### DGGE profile

**Figure 3:**
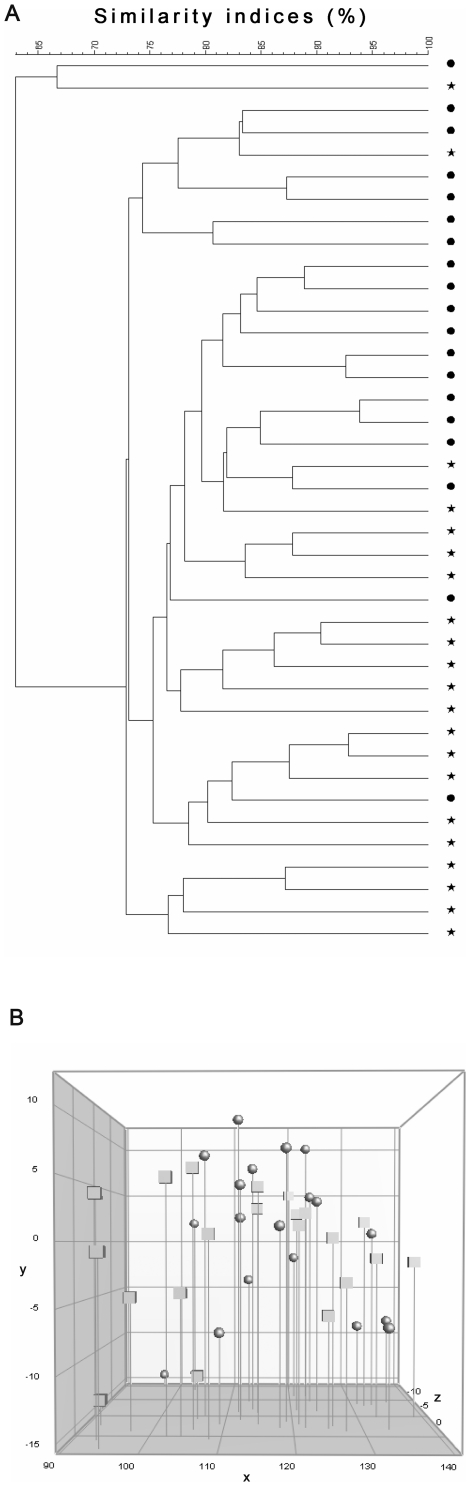
Dendrogram and Principal Component Analysis (PCA) based on Denaturing Gradient Gel Electrophoresis (DGGE) profiles from caecum samples. A) Dendrogram based on DGGE profiles of PCR amplified 16S rRNA V3 gene region from caecum samples collected from the NOD/Bom mice included in the incidence study. The dendrogram was made using Dice's similarity coefficient and Unweighted Pair Group Method with Arithmetic mean (UPGMA). The scale bar represents the similarity index in percent (100% indicate complete similarity 0% indicate complete dissimilarity). Similarities beyond 92% are defined as being incidental illustrated with a cut of line at 92%. Caecum sample analysis from each individual mouse is marked with either a dot or a star. Stars represent mice from the group of animals treated with 5% ethanol in the drinking water and dots represent mice from the control group receiving pure drinking water. B) Three-dimensional PCA based on DGGE profiles of PCR amplified 16S rRNA V3 gene region from caecum samples collected from the NOD/Bom mice included in the incidence study. Caecum samples representing the individual mice are marked with either a dot or a square. Squares represent mice treated with 5% ethanol in the drinking water and dots represent mice receiving pure drinking water. No grouping according to treatment was observed.

Based on caecum samples collected from the animals, DGGE was performed and a dendogram derived from the similarity of the DGGE pattern among samples (Fig. 3A) and a three-dimensional principal component analysis (3D-PCA) (Fig. 3B). The administration of alcohol did not significantly change the composition of the gut microbiota. DGGE dendograms revealed nine subclusters based on a chosen cut-off similarity level of 77% (Fig. 3A). The overall pattern in the three-dimensional principal component analysis (3D-PCA) (Fig. 3B) did not show a significant clustering referring to a higher similarity in the composition of the gut microbiota for the group of animals getting alcohol in their drinking water compared to the group of animals getting no alcohol. Also, the cluster pattern did not show a correlation to the week of diabetes onset (not shown).

## Discussion

The present study shows that the stimulatory effect of αGalCer on NKT cells is improved when alcohol is present during incubation with CD1 presenting cells. Conceivably, this is an effect on the loading of the CD1d molecules, as ethanol facilitated the passive loading of ^3^H-αGalCer in CD1d-expressing HeLa-D cells. Internalization of CD1d from the plasma membrane traffics through the early and late endosomes and lysosomal compartment where it interacts with MHC II invariant chain [20]; thus loading of CD1d is not restricted to the cell surface. Alcohol is taken up by the cells together with glycolipids through endocytosis possibly maintaining the function of solvent during trafficking to the endosomal compartment, hence alcohol facilitated antigen loading may occur both in endosomes and at the cell surface.

Interestingly, the alcohol effect was seen only at ethanol concentrations of 0.6% or more. While higher amounts of alcohol in the blood would be dangerous for most people, the same concentrations would likely be found in the intestine after normal alcohol consumption. Thus, the local levels in the intestine of alcohol consumption may be comparable to those used *in vitro*. This is of significance since NKT cell are active in the intestinal milieu which plays a role for the diabetes development [21].

NOD mice receiving 5% alcohol in their drinking water display a reduced diabetes incidence and lower blood glucose values at diagnosis. Ethanol given in 5% doses was well tolerated and did not affect behaviour. In contrast, when mice became diabetic they got more anxious and frightened, moved less, spent less time on the open arms or in the centre of the arena. Such behaviour corresponds to the natural behaviour of prey animal.

The dose of ethanol chosen was a compromise avoiding obvious ethanol influence on the liver. Due to the pre-test study the 5% alcohol dosage proved to fulfil this criterion, while this dose still represents a considerable intake of alcohol at least compared to man even after correction for body size. Yet, the ethanol concentration in blood was remarkably low, implying a relatively high ability of NOD mice to detoxify ethanol compared to humans.

The start of the study to 6 weeks of age was chosen because evidence of development of insulin resistance exists after treatment with alcohol in rats of younger age [22]. It is important for several treatments for diabetes reduction in NOD mice to start just after weaning or even earlier in life [23]. These considerations have to be compromised. However, a stronger effect of ethanol on diabetes incidence when given earlier in life cannot be ruled out by our present data.

In Western culture consumption of alcohol is relatively high, but no clear north-south gradient is seen in Europe as it is for the incidence of T1D [24]. T1D commonly develops in children not consuming alcohol themselves. Even in case this disease is manifests during adolescence of adulthood, the initiation of the disease process conceivably occurred before the age of alcohol introduction. Obviously, we are not proposing alcohol to be used as a prophylactic treatment against T1D. Nevertheless, mechanistically the findings are of interest. Indeed, in another autoimmune disorder, rheumatoid arthritis, mainly occurring in adults, alcohol has been associated with a decreased risk of developing the disease [25].

The similarity indices between DGGE profiles based on caecum samples collected from the animals at the time of diagnosed diabetes or at the end of the study indicates that the administration of alcohol at the age of 6 weeks, does not change the composition of the gut microbiota significantly at the time of sample collection. The decreased diabetic incidence cannot be directly referred to a difference in the overall composition of the gut microbiota between the animals.

Since ethanol does not facilitate insulin sensitivity in rats, but rather induces the opposite (resistance) [7], we doubt that the beneficial effect of ethanol has metabolic causes, for example by inducing a relative beta-cell rest. Considering an immunological mechanism modulated by ethanol seems therefore to be more pertinent. Diabetes is significantly reduced or delayed by increase of regulator CD4+CD25+ T cells or NKT cells. In NOD mice, the low level of NKT cells in particular seems to be of interest [26]. Also, patients developing T1D were shown to display low NKT cell levels [27], [28]. NKT cells are induced by stimulation with glycolipids presented by CD1d molecules. Interestingly, CD1d knock-out NOD mice show an exacerbation of diabetes [29], whereas upregulation of CD1d expression within the beta cells restores the immune regularity function of NKT cells preventing diabetes [30].

In two mice strains the levels of some glycosphingolipids in pancreas have been examined: sulfatide is significantly more present in NOD compared to BALB/c mice, and sulfated lactosyl ceramide is expressed in NOD mice only [31].

We favour the interpretation that alcohol results in improved glycolipid loading to CD1d and signalling to NKT cells, affecting development of experimental autoimmune diabetes. This is supported by significantly increased numbers of NKT cells after alcohol intake. NKT cells can inhibit diabetes onset by impairing the development of pathogenic T cells specific for pancreatic beta cells [32]. Additional effects that may act in concert cannot be excluded; alcohol impairs cytokine-driven differentiation and function of myeloid and plasmacytoid dendritic cells *in vitro* [33]. NKT cells are potent regulators that can inhibit development of T1D by impairing the differentiation of anti-islet T cells into Th1 effector cells, and for doing this cell contact seems to be crucial [34]. However still for raising the NKT cells the CD1d presentation is decisive [12].
